# DNA methylation and gene expression of *TXNIP* in adult offspring of women with diabetes in pregnancy

**DOI:** 10.1371/journal.pone.0187038

**Published:** 2017-10-27

**Authors:** Azadeh Houshmand-Oeregaard, Line Hjort, Louise Kelstrup, Ninna S. Hansen, Christa Broholm, Linn Gillberg, Tine D. Clausen, Elisabeth R. Mathiesen, Peter Damm, Allan Vaag

**Affiliations:** 1 Center for Pregnant Women with Diabetes, Department of Obstetrics, Rigshospitalet, Copenhagen, Denmark; 2 Diabetes and Metabolism, Department of Endocrinology, Rigshospitalet, Copenhagen, Denmark; 3 Institute of Clinical Medicine, Faculty of Health and Medical Sciences, University of Copenhagen, Copenhagen, Denmark; 4 Danish PhD School of Molecular Metabolism/Danish Diabetes Academy, Odense, Denmark; 5 Department of Gynecology and Obstetrics, Nordsjaellands Hospital, University of Copenhagen, Hilleroed, Denmark; 6 Center for Pregnant Women with Diabetes, Department of Endocrinology, Rigshospitalet, Copenhagen, Denmark; University of Texas Health Science Center at San Antonio, UNITED STATES

## Abstract

**Background:**

Fetal exposure to maternal diabetes increases the risk of type 2 diabetes (T2DM), possibly mediated by epigenetic mechanisms. Low blood *TXNIP* DNA methylation has been associated with elevated glucose levels and risk of T2DM, and increased skeletal muscle *TXNIP* gene expression was reported in subjects with impaired glucose metabolism or T2DM. Subcutaneous adipose tissue (SAT) and skeletal muscle play a key role in the control of whole body glucose metabolism and insulin action. The extent to which *TXNIP* DNA methylation levels are decreased and/or gene expression levels increased in SAT or skeletal muscle of a developmentally programmed at-risk population is unknown.

**Objective and methods:**

The objective of this study was to investigate *TXNIP* DNA methylation and gene expression in SAT and skeletal muscle, and DNA methylation in blood, from adult offspring of women with gestational diabetes (O-GDM, n = 82) or type 1 diabetes (O-T1DM, n = 67) in pregnancy compared with offspring of women from the background population (O-BP, n = 57).

**Results:**

SAT *TXNIP* DNA methylation was increased (p = 0.032) and gene expression decreased (p = 0.001) in O-GDM, but these differences were attenuated after adjustment for confounders. Neither blood/muscle *TXNIP* DNA methylation nor muscle gene expression differed between groups.

**Conclusion:**

We found no evidence of decreased *TXNIP* DNA methylation or increased gene expression in metabolic target tissues of offspring exposed to maternal diabetes. Further studies are needed to confirm and understand the paradoxical SAT *TXNIP* DNA methylation and gene expression changes in O-GDM subjects.

## Introduction

Fetal exposure to maternal diabetes is associated with increased risk of metabolic disease in offspring, and this association is greater than the expected genetic risk associated with a family history of diabetes [1–5]. Increasing evidence indicates that epigenetic mechanisms play a role in mediating this increased risk of metabolic disease associated with fetal exposure to diabetes in pregnancy [6–10].

Addition of methyl groups at cytosine-guanine dinucleotides (CpGs) in regulatory/promoter regions in DNA, known as DNA methylation, typically leads to transcriptional repression and decreased expression of the gene in question [11]. Thioredoxin-interacting protein (*TXNIP*) is a ubiquitously expressed protein that inhibits the key cellular antioxidant protein thioredoxin, and thus regulates the cellular redox state, promoting oxidative stress and apoptosis [12, 13]. A high *TXNIP* DNA methylation percentage at CpG site cg19693031 in blood has consistently been associated with lower fasting blood glucose, HbA1c and/or HOMA-IR levels, as well as a lower prevalence and a decreased risk of type 2 diabetes (T2DM) in different studies [14–19].

*TXNIP* gene expression is stimulated by glucose and suppressed by insulin in a variety of cells and tissues, including skeletal muscle and adipose tissue and cells [20–22], and *TXNIP* gene expression is upregulated in skeletal muscle samples from subjects with diabetes and prediabetes [22]. Increased *TXNIP* expression inhibits insulin-mediated glucose uptake in skeletal muscle and adipocytes, while *TXNIP* inhibition increases glucose uptake [22, 23]. Thus, *TXNIP* is a glucose and insulin-sensitive switch regulating glucose metabolism by controlling glucose uptake in the periphery.

Altogether, the abovementioned results implicate altered *TXNIP* DNA methylation and/or expression as a potential pathogenic mechanism in the development of T2DM. However, almost all studies of *TXNIP* DNA methylation are performed on circulating blood cells [14, 16, 18, 19], and information on methylation in other metabolically relevant tissues such as fat and skeletal muscle is lacking. Moreover, direct evidence of epigenetic changes caused by fetal programming of diabetes is scarce, with only a few target genes identified in metabolically active tissues. We have access to metabolically active tissues in a unique cohort of subjects exposed to maternal diabetes in pregnancy, and with a known predisposition to T2DM.

The aim of our study was to investigate whether exposure to intrauterine hyperglycemia may be associated with changes in *TXNIP* DNA methylation in subcutaneous adipose tissue (SAT), skeletal muscle and blood as well as *TXNIP* gene expression levels in SAT and skeletal muscle. Based on previous studies showing an increased risk of T2DM in offspring exposed to intrauterine hyperglycemia, we expected decreased *TXNIP* DNA methylation in SAT, skeletal muscle and blood, and conversely increased *TXNIP* gene expression levels in SAT and skeletal muscle in offspring exposed to maternal diabetes compared to controls.

## Methods

### Study design and setting

**Fig 1:**
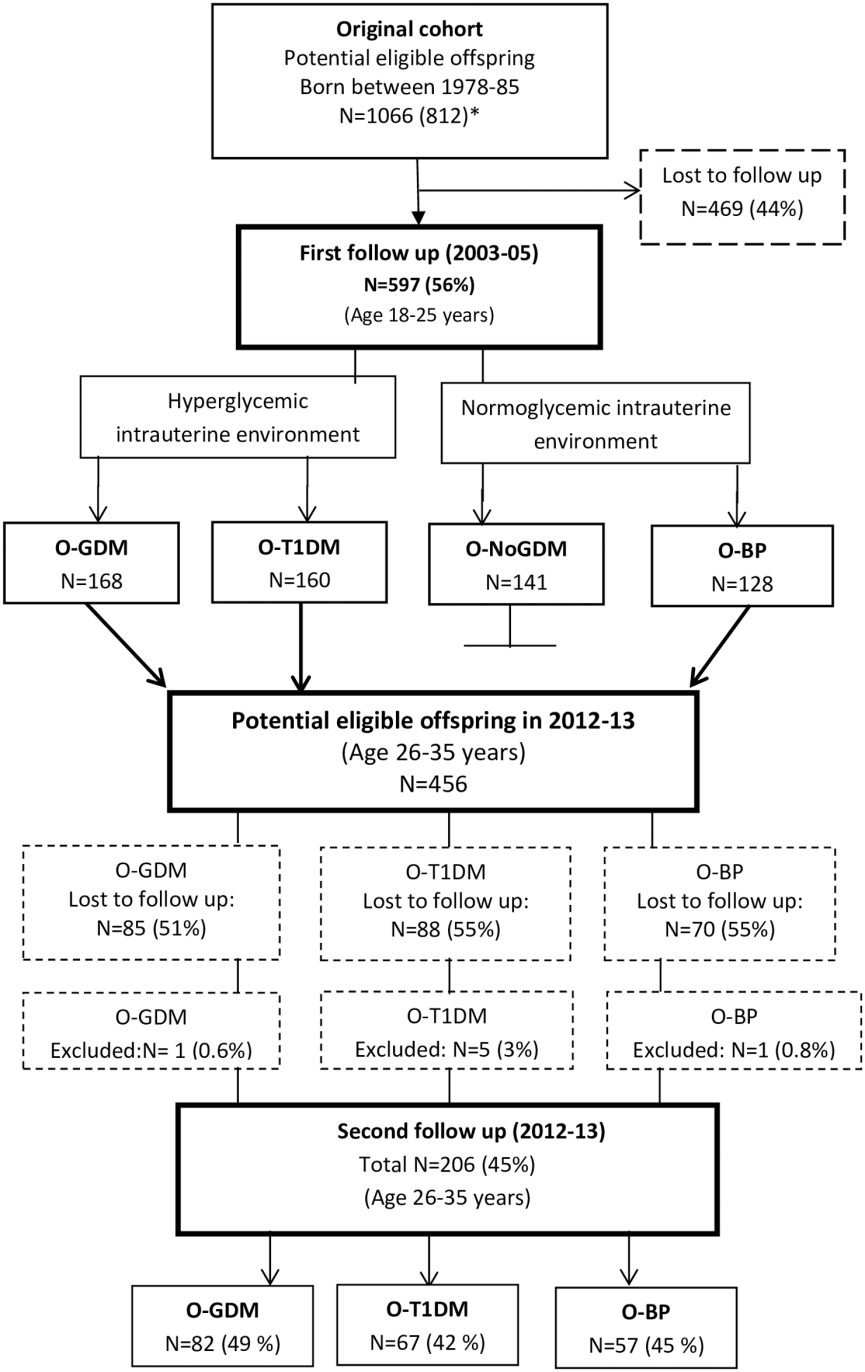
Study design- subjects participating and lost to follow-up. O-BP: offspring of women from the background population; O-GDM: offspring of women with gestational diabetes; O-NoGDM: offspring of women with risk factors for gestational diabetes but normal glucose tolerance in pregnancy (not invited to participate in second round of follow-up); O-T1DM: offspring of women with type 1 diabetes in pregnancy. *O-NoGDM (n = 254) were not invited to participate in the second round of follow-up, leaving 812 eligible offspring from the original cohort (1066–254 = 812) divided into the three groups O-GDM, O-T1DM and O-BP.

The current study is the second follow-up in an observational study of a birth cohort exposed to diabetes in pregnancy. Material from the same cohort has been used previously, and study design, inclusion criteria and baseline data have been described in detail elsewhere [1, 2, 24, 25]. The participants were adult offspring of women with either gestational diabetes (O-GDM, N = 82) or type 1 diabetes (O-T1DM, N = 67) in pregnancy, and a randomly selected control group consisting of offspring of women from the background population (O-BP, N = 57). Participants were born in the period between 1978–1985 at Rigshospitalet, in Copenhagen, Denmark, and were between 26–35 years of age at the time of examination. The original cohort consisted of 1066 offspring. Of these 254 belonged to a group that was not re-invited for the second follow-up, leaving a total of 812 potentially eligible offspring in the original cohort, 597 of whom participated in the first cross-sectional follow-up study in 2003–2005 [1, 2, 24]. Of these, 456 participants were eligible for participation in the second follow-up, and 250 were lost or excluded for various reasons, resulting in a total of 206 participants (25% of the original cohort or 45% from the first follow-up in the current study as previously described [24, 25] (Fig 1).

### Diabetes in pregnancy in Denmark during the period of 1978–1985: Maternal selection criteria

In the abovementioned period in Denmark, screening for gestational diabetes in pregnancy was based on the presence of one or more risk factors and two consecutive fasting blood glucose values ≥ 4.1 mmol/l. The definitive GDM diagnosis was then confirmed by a 3-hour 50-g oral glucose tolerance test (OGTT), which was defined as abnormal if two or more blood glucose values exceeded the mean +3 SD of a standard reference curve for a group of healthy, normal-weight, non-pregnant women without a family history of diabetes [26]. The mean + 3SD for venous plasma glucose values were 6.4 mmol/l at t = 0, 10.1 mmol/l at t = 30 min, 7.6 mmol/l at both t = 120 and 150 min, and 6.6 mmol/l at t = 180 min [27]. Only women with diet-treated GDM were included, in order to minimize potential risk of misclassification from other types of diabetes (e.g. early stage T1DM, undiagnosed T2DM and MODY).

Mothers with type 1 diabetes fulfilled the following criteria: onset of diabetes at age 40 or younger, classical history of hyperglycemia symptoms before diagnosis, and insulin treatment started 6 months or less after diagnosis.

Mothers from the background population were unselected women routinely referred to Rigshospitalet for antenatal care and delivery in the same period (1978–1985) [2]. They were identified from maternal medical records of all deliveries in the study period and sampled consecutively according to maternal day of birth

### Outcome variables

Outcomes of interest were *TXNIP* DNA methylation in SAT, skeletal muscle and blood, and gene expression in SAT and skeletal muscle in offspring exposed to maternal diabetes compared to unexposed offspring. Furthermore, we studied associations between maternal blood glucose levels on one hand, and offspring *TXNIP* DNA methylation or gene expression on the other hand, as well as correlations between offspring *TXNIP* DNA methylation and gene expression levels and markers of offspring glucose and insulin sensitivity.

### Exposure variables

Exposure to maternal diet-treated GDM or T1DM, as determined by offspring group, was the primary exposure variable. GDM mothers underwent OGTT’s during pregnancy and information regarding fasting and 2-hour blood glucose levels was available for these women. The procedure for T1DM pregnancies in the baseline period involved hospitalization in the 1^st^ and 3^rd^ trimester with measurement of blood glucose 7 times a day for 3 days, and mean glucose values were calculated from these 3-day profiles in the 1^st^ and 3^rd^ pregnancy trimester. Maternal pregnancy blood glucose values for GDM and T1DM mothers were thus also used as exposure variables.

### Confounders

Multivariate regression analyses were performed to strengthen the hypothesis that observed differences in *TXNIP* DNA methylation and gene expression between the exposure and control groups were due to exposure to maternal diabetes. Maternal pre-pregnancy BMI, age at delivery, smoking status, family history of diabetes, as well as offspring gender and age at follow-up were used as potential confounders in regression analyses.

### Examination of participants at follow-up

The participants were recruited and examined in the period between May 2012 and September 2013. After an overnight fast, they underwent SAT biopsies from the abdomen and skeletal muscle biopsies from the vastus lateralis muscle of the thigh using a Bergstrom needle. A total of 70–300 mg adipose tissue and 50–200 mg skeletal muscle was obtained, and the tissue was immediately frozen in liquid nitrogen and stored at -80°C until analysis.

The biopsies were followed by a 2-hour, 75-g oral OGTT, and clinical examination with measurements of height, weight, waist and hip circumference, blood pressure, and a dual x-ray absorptiometry (DEXA) whole-body scanning (GE Medical Systems Lunar Prodigy Advance, Fairfield, Connecticut, USA) to determine body composition. Body fat percent (BF%) was defined as total fat mass divided by total body mass. Insulin resistance was calculated using homeostatic model assessment (HOMA-IR) [28] and glucose tolerance was assessed according to WHO criteria of 2006 [29].

The study was in accordance with the Declaration of Helsinki and approved by the Danish National Committee on Health Research Ethics. All subjects received written and oral information and provided written consent before participation.

### DNA methylation

We extracted genomic DNA from the skeletal muscle biopsies using the DNeasy blood and tissue Kit, from the SAT biopsies using the QIAamp DNA Mini Kit, and from blood (buffy coats) using the QIAamp 96 DNA blood kit (all Qiagen). A total of 10–40 mg SAT and skeletal muscle tissue was used to extract 500 ng (skeletal muscle and blood) and 400 ng (SAT) DNA, respectively, which was then bisulfite converted using the EpiTect Bisulfite Kit (Qiagen). DNA methylation was then measured at CpG site cg19693031, located in the 3’UTR region approximately 3000 base pairs downstream from the *TXNIP* transcription start site, using bisulfite pyrosequencing. Methylation differences at this CpG site may alter the binding affinity of methylation-sensitive transcription regulators [16]. PyroMark Assay Design 2.0 software was used to design primers, and pyrosequencing of the PCR products was performed with the PyroMarkQ96 instrument (Qiagen). Primer sequences were as follows: forward primer, 5´-TGTTTGTTGGATGGGTTTAAAAATAATT-3´, reverse primer (biotinylated), 5´-AAACCTCCAAAAAACCTTAAAAAACTT-3´, sequencing primer, 5´GGGTTAGGTAAAAATGG -3´.

### Gene expression

20–90 mg SAT and skeletal muscle tissue was used to extract 400 ng RNA using the miRNEasy Mini Kit (Qiagen) for SAT and the Trizol method for skeletal muscle. RNA concentrations were measured using a NanoDrop 1000 spectrophotometer (Thermo Scientific). RNA was synthesized to cDNA using the QuantiTect Reverse Transcription Kit (Qiagen) using random primers. Target-specific *TXNIP* gene primers were then used to amplify the TXNIP gene from our cDNA. These were designed using human specific databases (Ensembl Genome Browser) and Universal Probe Library (Roche Applied Science). The primer sequences were: forward primer, 5´-GGCTAAAGTGCTTTGGATGC-3´, and reverse primer: 5´-AGGTCTCATGATCACCATCTCA-3´. Primers were synthesized by DNA Technology and optimization was performed before use to determine primer working concentrations. *TXNIP* mRNA expression levels were evaluated in duplicates using SYBR Green mastermix by reverse transcription quantitative PCR (RT qPCR) using the ABI PRISM 7900 ViiA7 Real-Time PCR System (Applied Biosystems), where the PCR products are measured as they accumulate in “real-time”. Real-time qPCR is a highly sensitive and specific technique, and is considered to be the gold standard by which a very low number of RNA molecules can be detected. SYBR Green dye has the potential to generate false positive signals as it can bind to any double-stranded DNA in the sample (for example primer-dimers), and to overcome this problem we included non-reverse transcriptase controls to test for the presence of contaminating DNA, and we tested for the presence of primer-dimers by melting-curve analysis where we accepted primers with only one top. We measured mRNA expression in duplicates to take technical variation into account, and a variation of up to 5% was accepted between the two values, as per standard recommendation and protocol [30]. The variation was below 1% for all mRNA samples in this study. Fold changes in mRNA expression levels were calculated after normalization to the hypoxanthine phosphoribosyl transferase 1 (*HPRT1)* reference gene (forward primer: 5´-TGACCTTGATTTATTTTGCATACC-3´, reverse primer: 5´-CGAGCAAGACGTTCAGTCCT-3´).

### Statistics

All statistical analyses were performed using IBM SPSS statistics version 22. All comparisons were to the O-BP control group, performed using independent samples Student’s t-test for comparison of means between continuous variables or chi-squared test for comparison of proportions for categorical variables. Nonparametric data was log-transformed prior to entering statistical analysis. Forced-entry multivariate linear regression analysis was used to examine the impact of maternal metabolic disease on offspring DNA methylation and gene expression. We used two models—in model 1 we corrected for maternal pre-pregnancy BMI, age at delivery, smoking status, family history of diabetes, as well as offspring gender and age. Model 2 was a post-hoc analysis in which we added offspring HOMA-IR, HbA1c and total body fat % as potential mediators, as these have been shown to be associated with *TXNIP* DNA methylation and gene expression. Missing values were excluded listwise (complete case analyses) in regression analyses. *TXNIP* expression levels were log transformed prior to linear regression analysis to meet assumptions of homoscedasticity. A two-sided p-value<0.05 was considered statistically significant for all analyses

## Results

### Baseline clinical data

Characteristics of the study population and maternal data have been previously published [24] and are shown in Table 1. Offspring exposed to maternal diabetes had significantly higher 2-hour OGTT glucose values, and for O-GDM, higher 30-minute glucose and HbA1c values. There were no other differences in the majority of baseline and anthropometric data between the two exposure groups and the control group. The GDM women had a significantly higher age at delivery, higher pregestational BMI, and a higher proportion of these women were smokers and had pregestational overweight compared to the women from the background population, as previously described [24]

**Table 1:** Baseline clinical characteristics in adult offspring of women with gestational diabetes (O-GDM) or type 1 diabetes (O-T1DM) compared to offspring of women from the background population (O-BP).

	O-GDM	O-T1DM	O-BP	O-GDM vs. O-BP *p-value*	O-T1DM vs. O-BP *p-value*
**N (Total = 206)**	82	67	57		
**Maternal data (1978–85)**					
**Age at delivery (years)**	30.4(5.2)	26.4 (4.7)	26.8 (4.6)	**<0.001**	0.645
**Pregestational BMI (kg/m2)**	24.3 (5.6)	21.7 (1.9)	21.2 (3.5)	**<0.001**	0.301
**Pregestational overweight (BMI ≥ 25 kg/m2)**	34% (28/82)	8% (5/64)	12% (7/57)	**0.003**	0.412
**Family history of diabetes (yes vs. no)**	26% (21/82)	25% (17/67)	16% (9/57)	0.166	0.191
Smoking status (yes vs. no)*	32% (22/69)	63% (37/59)	58% (26/45)	**0.006**	0.610
**Fasting blood glucose before OGTT (mmol/l)**	5.2 (0.6)	NA	NA	NA	NA
**120 min blood glucose during OGTT (mmol/l)**	7.9 (1.8)	NA	NA	NA	NA
**Mean blood glucose in first trimester**	NA	9.0 (3.0)	NA	NA	NA
**Mean blood glucose in third trimester**	NA	6.7 (1.6)	NA	NA	NA
**Offspring anthropometric data**					
**Age (year)**	30.2 (2.1)	30.8 (2.4)	30.8 (2.4)	0.183	0.879
**Gender (male)**	52% (43/82)	46% (31/67)	46% (26/57)	0.429	0.942
**Weight (kg)**	77.8 (17.4)	78.3 (17.9)	75.3 (16.5)	0.398	0.331
**Height (meter)**	1.76 (0.10)	1.74 (0.10)	1.74 (0.10)	0.481	0.676
**Total body fat (%)**	29.8% (0.1)	31.4% (0.1)	28.7% (0.1)	0.428	0.093
**BMI (kg/m**^**2**^**)**	25.2 (5.1)	26.0 (5.9)	24.6 (3.9)	0.493	0.113
**Obese (BMI ≥ 30 kg/m**^**2**^**)**	15% (12/82)	16% (11/67)	7% (4/57)	0.166	0.110
**Results of OGTT**^a^					
**Fasting plasma glucose (mmol/l) "door step"**	5.0 (0.7)	4.9 (0.4)	4.9 (0.3)	0.245	0.381
**30 min. plasma glucose (mmol/l)**	8.2 (1.7)	7.8 (1.7)	7.3 (1.6)	**0.006**	0.125
**120-min plasma glucose (mmol/l)**	6.0 (1.8)	6.3 (1.7)	5.3 (1.2)	**0.016**	**0.001**
**HbA1C_IFCC (mmol/mol)**	35.1 (3.6)	34.5 (3.3)	34.0 (2.8)	0.076	0.415
**HbA1C_DCCT (%)**	5.4 (0.3)	5.3 (0.3)	5.3 (0.3)	0.079	0.569
**Abnormal glucose tolerance (IFG, IGT or T2DM)**	13% (11/82)	13% (9/67)	5% (3/57)	0.116	0.125
**IFG**	1% (1/82)	0% (0/67)	0% (0/57)	0.403	NA
**IGT**	7% (6/82)	10% (7/67)	5% (3/57)	0.628	0.291
**Both IFG and IGT**	1% (1/82)	0%(0/67)	0% (0/57)	0.403	NA
**Pre-diabetes (IFG and/or IGT)**	10% (8/82)	10% (7/67)	5% (3/57)	0.334	0.291
**T2DM (diagnosed at follow-up)**	2% (2/82)	1.5% (1/67)	0% (0/57)	0.235	0.354
**T2DM (previously known)**	1% (1/82)	1.5%(1/67)	0% (0/57)	0.403	0.354
**HOMA-IR**^b^	1.77 (1.56–2.02)	1.95 (1.71–2.22)	1.72 (1.47–2.02)	0.784	0.222
**Plasma samples**^b^					
**Fasting insulin (pmol/l)**	49 (43–55)	54 (48–61)	49 (42–56)	0.953	0.255
**120 min. insulin (pmol/l)**	252 (210–304)	281 (240–329)	223 (185–268)	0.350	0.056
**Triglycerides (mmol/l)**	0.89 (0.81–0.98)	0.84 (0.76–0.93)	1.00 (0.76–1.31)	0.391	0.233
**LDL-cholesterol (mmol/l)**	2.72 (2.57–2.87)	2.64 (2.48–2.81)	2.79 (2.60–3.00)	0.540	0.233
**HDL-cholesterol (mmol/l)**	1.33 (1.26–1.41)	1.44 (1.37–1.52)	1.36 (1.26–1.48)	0.605	0.241
**Total cholesterol (mmol/l)**	4.68 (4.51–4.84)	4.70 (4.53–4.89)	4.78 (4.56–5.02)	0.447	0.592
**Hs-CRP(mg/l)**	1.02 (0.81–1.28)	1.17 (0.90–1.52)	0.87 (0.66–1.14)	0.369	0.124

Data is mean (SD), median (25th–75th percentiles) or percentage (number), unless otherwise indicated.

All comparisons are to the O-BP control group. Analysis of differences (means or proportions) between groups performed by independent samples t-test or X^2-^ test, respectively. P-values < 0.05 are bold. Total body fat % = (total fat mass/total body mass)x100.

*Data on smoking status was missing for 33 subjects

^a^. Based on 2-hour 75g OGTT and evaluated according to WHO-criteria of 2006 (ref. [30])

^b^. Data is presented as geometric mean and 95% confidence intervals

**Abbreviations:** BMI: body mass index; HDL: high density lipoprotein; HOMA-IR: homeostatic model assessment insulin resistance; Hs-CRP: high sensitivity C-reactive protein; IFG: impaired fasting glucose; IGT: impaired glucose tolerance; LDL: low density lipoprotein; NA: not applicable; OGTT: oral glucose tolerance; T1DM: type 1 diabetes; T2DM: type 2 diabetes

### *TXNIP* DNA methylation and gene expression in subcutaneous adipose tissue

**Fig 2:**
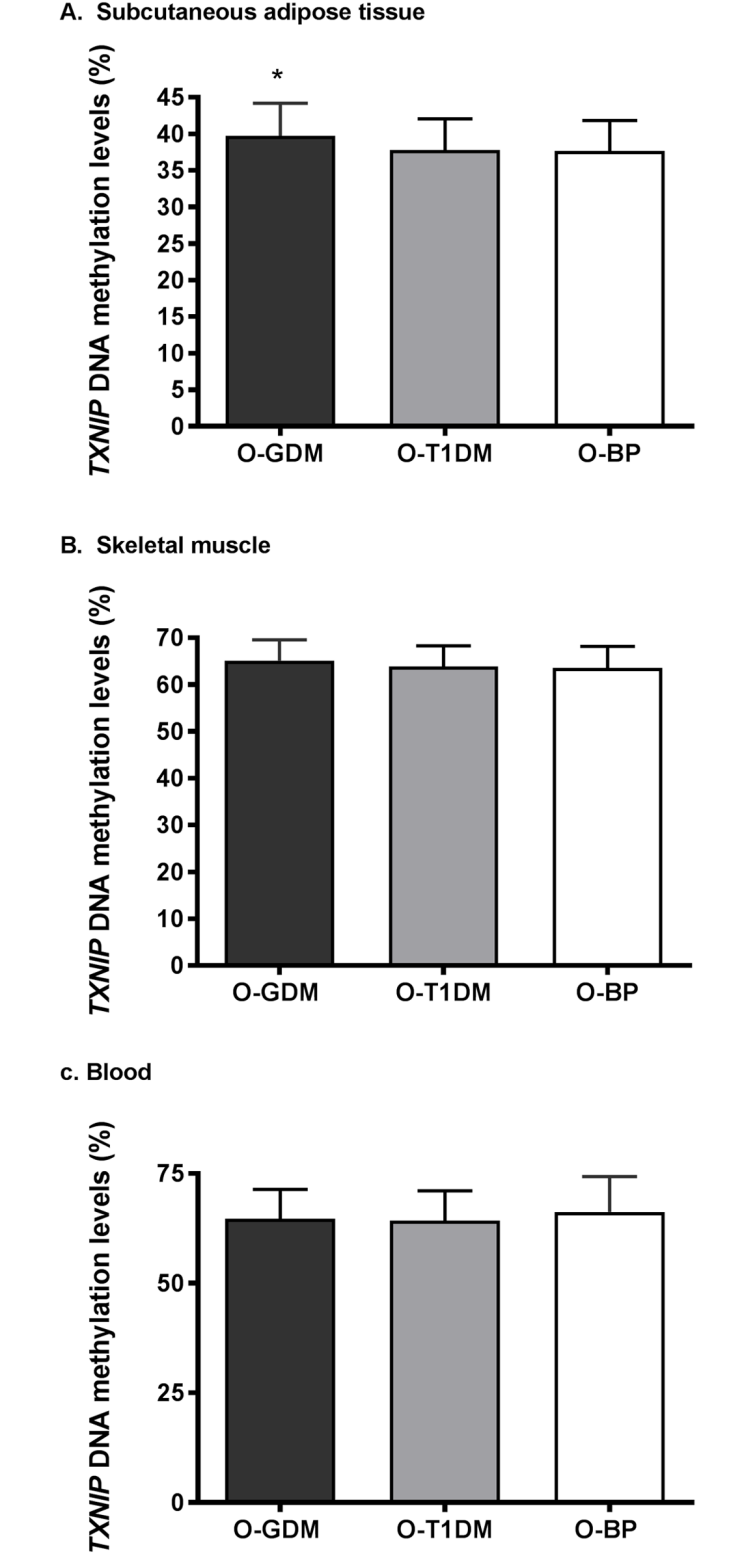
*TXNIP* DNA methylation in A. subcutaneous adipose tissue, B. skeletal muscle and C. blood from offspring of women with gestational diabetes (O-GDM), offspring of women with type 1 diabetes (O-T1DM) in pregnancy, and offspring of women from the background population (O-BP). Data is presented as mean +/- SD. Differences between mean of exposure and control groups calculated using independent samples t-test. All comparisons are to the O-BP control group. DNA methylation was measured at the CpG site cg19693031. * P<0.05.

In univariate analyses, *TXNIP* DNA methylation in SAT was increased in O-GDM compared to O-BP (p = 0.032) (Fig 2A). This difference was attenuated after adjustment for confounders in model 1 (p = 0.063) and additional adjustment for mediators in model 2 (p = 0.166). There was no difference in DNA methylation levels between O-T1DM and O-BP (Table 2).

**Table 2:** *TXNIP* DNA methylation and gene expression in subcutaneous adipose tissue, skeletal muscle and blood from offspring of women with gestational diabetes (O-GDM) or type 1 diabetes (O-T1DM) compared to offspring of women from the background population (O-BP) in univariate and multivariate analyses.

	O-GDM	O-T1D	O-BP	O-GDM vs. O-BP p-value	O-T1DM vs. O-BP p-value
***TXNIP* methylation subcutaneous adipose tissue (%);** O-GDM N = 53; O-T1DM N = 49; O-BP N = 35	39.73 (4.48)	37.76 (4.29)	37.66 (4.18)	**0.032** Model 1 0.063 Model 2 0.166	0.909 Model 1 0.336 Model 2 0.419
***TXNIP* methylation skeletal muscle (%)** O-GDM N = 62; O-T1DM N = 62; O-BP N = 41	65.14 (4.42)	63.91 (4.39)	63.56 (4.59)	0.084 Model 1 0.188 Model 2 0.369	0.697 Model 1 0.369 Model 2 0.440
***TXNIP* methylation blood (%)** O-GDM N = 82; O-T1DM N = 65; O-BP N = 57	64.75 (6.66)	64.27 (6.84)	66.25 (8.07)	0.232 Model 1 0.549 Model 2 0.627	0.144 Model 1 0.430 Model 2 0.511
***TXNIP* expression subcutaneous adipose tissue (arbitrary units)**^a^ O-GDM N = 58; O-T1DM N = 59; O-BP N = 42	1.29 (0.56)	1.53 (0.76)	1.91 (1.25)	**0.001** Model 1 **0.024** Model 2 0.080	0.058 Model 1 0.271 Model 2 0.668
***TXNIP* expression skeletal muscle (arbitrary units)**^a^ O-GDM N = 76; O-T1DM N = 63; O-BP N = 42	1.09 (0.47)	1.09 (0.48)	1.06 (0.45)	0.800 Model 1 0.574 Model 2 0.722	0.786 Model 1 0.790 Model 2 0.751

Data is mean (SD). All comparisons are to the O-BP control group. Analysis of differences (means or proportions) between groups was performed by independent samples t-test.

p-values < 0.05 are bold

*TXNIP* expression is calculated relative to the HPRT reference gene.

^a^ Data was log transformed prior to t-test.

Model 1: adjusted for maternal pre-pregnancy BMI, age at delivery, smoking status, family history of diabetes, and offspring gender and age at follow-up

Model 2: model 1 with additional adjustment for offspring HOMA-IR, Hba1c, and total body fat percent.

**Fig 3:**
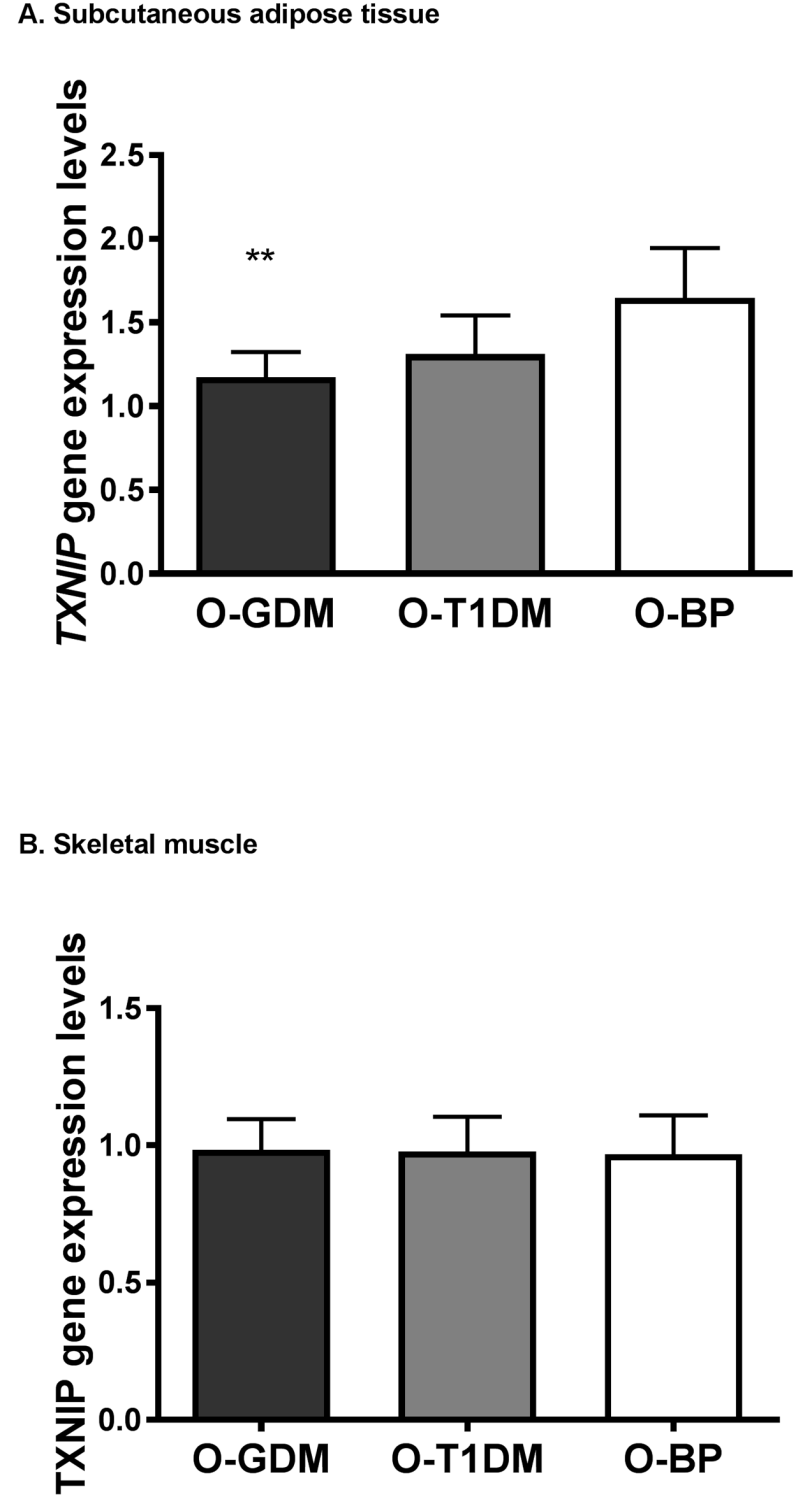
*TXNIP* gene expression in A. subcutaneous adipose tissue and B. skeletal muscle from offspring of women with gestational diabetes (O-GDM), offspring of women with type 1 diabetes (O-T1DM) in pregnancy, and offspring of women from the background population (O-BP). Data is presented as geometric mean with 95% confidence intervals. Differences between mean of exposure and control groups calculated using independent samples t-test. All comparisons are to the O-BP control group. Gene expression levels are shown relative to the HPRT reference gene. **P<0.01.

*TXNIP* expression was decreased in SAT from O-GDM (p = 0.001) and near-significantly decreased in O-T1DM (p = 0.058) compared to O-BP (Fig 3A). Adjusting for confounders (model 1), maternal GDM remained negatively associated with SAT *TXNIP* expression (p = 0.024), while this association was attenuated when further adjusted for mediators in model 2 (0 = 0.080) (Table 2).

### *TXNIP* DNA methylation and gene expression in skeletal muscle

There were no significant differences in skeletal muscle *TXNIP* DNA methylation or expression between groups (Figs 2B and 3B, Table 2).

### *TXNIP* DNA methylation in blood

There were no differences in blood *TXNIP* DNA methylation between the groups (Fig 2C, Table 2).

### Association between maternal blood glucose levels and offspring *TXNIP* DNA methylation and gene expression

Maternal blood glucose values (fasting or 2-hour for women with GDM and mean blood glucose values in 1^st^ or 3^rd^ trimester for women with T1DM) were not associated with *TXNIP* DNA methylation in SAT, skeletal muscle or blood in neither univariate nor multivariate linear regression analyses. Maternal 1^st^ trimester blood glucose values were significantly negatively associated with SAT *TXNIP* gene expression in model 1 (p = 0.011) and remained significant in model 2 (p = 0.024).

### Correlations between *TXNIP* DNA methylation and offspring markers of glucose and insulin sensitivity in the cohort as a whole

There was a negative association between SAT *TXNIP* DNA methylation and offspring 120-min plasma glucose values and total body fat %. Skeletal muscle *TXNIP* DNA methylation was negatively associated with offspring fasting plasma glucose only. Blood *TXNIP* DNA methylation was significantly negatively associated with offspring fasting plasma glucose and insulin and HOMA-IR (Table 3). Although associations were attenuated, the patterns in associations in the offspring groups individually were similar to those in the cohort as a whole (S1 Table).

**Table 3:** Correlations between *TXNIP* DNA methylation and gene expression and offspring markers of metabolic disease.

METHYLATION				GENE EXPRESSION	
	***TXNIP* DNA methylation SAT**	***TXNIP* DNA methylation skeletal muscle**	***TXNIP* DNA methylation blood**	***TXNIP* gene expression SAT**	***TXNIP* gene expression skeletal muscle**
***TXNIP* DNA methylation SAT**		**0.282 (0.002)**	0.048 (0.576)	0.026 (0.791)^a^	**-0.225 (0.011)** ^a^
***TXNIP* DNA methylation skeletal muscle** **muscle**	**0.282 (0.002)**		**0.220 (0.005)**	0.004 (0.964) ^a^	-0.128 (0.104) ^a^
***TXNIP* DNA methylation blood**	0.048 (0.576)	**0.220 (0.005)**		0.090 (0.265) ^a^	0.061 (0.420) ^a^
***TXNIP* gene expression SAT**	0.026 (0.791)^a^	0.004 (0.964) ^a^	0.090 (0.265) ^a^		-0.092 (0.265) ^a^
***TXNIP* gene expression skeletal muscle**	**0.225 (0.011)** ^a^	-0.128 (0.104) ^a^	0.061 (0.420) ^a^	-0.092 (0.265) ^a^	
**Fasting plasma glucose (mmol/l)**	-0.104 (0.228)	**-0.177 (0.023)**	**-0.143 (0.041)**	**-0.176 (0.027)** ^a^	0.049 (0.516) ^a^
**120-min plasma glucose (mmol/l)**	**-0.222 (0.011)**	-0.108 (0.177)	0.011 (0.882)	-0.151 (0.065) ^a^	**0.272 (<0.001)** ^a^
**HbA1C DCCT (%)**	0.093 (0.279)	0.005 (0.946)	-0.103 (0.144)	**-0.211 (0.008)** ^a^	0.044 (0.554) ^a^
**HOMA-IR**	-0.040 (0.661)	-0.111 (0.181)	**-0.156 (0.035)**	**-0.408 (<0.001)** ^a^	**0.256 (0.001)** ^a^
**Fasting insulin (pmol/l)**	-0.021 (0.814)	-0.080 (0.334)	**-0.151 (0.041)**	**-0.400 (<0.001)** ^a^	**0.254 (0.001)** ^a^
**120-min insulin (pmol/l)**	-0.105 (0.244)	-0.081 (0.322)	-0.064 (0.385)	**-0.300 (<0.001)** ^a^	**0.409 (<0.001)** ^a^
**Total body fat (%)**	**-0.175 (0.041)**	-0.063 (0.426)	0.010 (0.882)	**-0.289 (<0.001)**	**0.349 (<0.001)**

Correlations are presented as Pearsons rank coefficient R (p-value) unless otherwise indicated. P-values <0.05 are bold.

^a^Spearman’s rank coefficient.

SAT: subcutaneous adipose tissue

HOMA-IR: homeostatic model assessment insulin resistance

### Correlations between *TXNIP* gene expression and offspring markers of glucose and insulin sensitivity in the cohort as a whole

There was a significant negative correlation between SAT *TXNIP* gene expression and offspring fasting plasma glucose, Hba1c, HOMA-IR, fasting and 2-hour OGTT plasma insulin levels as well as offspring total body fat percent. By contrast, there was a significant positive correlation between skeletal muscle *TXNIP* gene expression and offspring 2-hour OGTT plasma glucose levels, HOMA-IR, fasting and 2-hour OGTT insulin levels, and total body fat percent (Table 3). Again, similar patterns were seen when examining offspring groups individually, although attenuated (S1 Table).

### Differences in *TXNIP* DNA methylation and gene expression in offspring with prediabetes

We found significantly decreased SAT *TXNIP* DNA methylation (p = 0.031) and in skeletal muscle increased *TXNIP* gene expression (p = 0.038) and borderline decreased *TXNIP* DNA methylation (p = 0.070) in offspring with abnormal glucose tolerance (impaired fasting glucose IFT, impaired glucose tolerance IGT, and T2DM) compared to those with normal glucose tolerance (Table 4). There were no differences in blood *TXNIP* DNA methylation or SAT gene expression between offspring with abnormal glucose tolerance compared to offspring with normal glucose tolerance.

**Table 4:** *TXNIP* DNA methylation and gene expression in subjects with normal glucose tolerance compared to subjects with prediabetes.

	Abnormal OGTT (IFG, IGT, or T2DM)	Normal OGTT	Difference between subjects with normal and abnormal OGTT (p-value)
***TXNIP* DNA methylation SAT (N = 14/123)**	36.73 (2.81)	38.70 (4.53)	**0.031**
***TXNIP* DNA methylation skeletal muscle (N = 18/147)**	62.48 (3.91)	64.50 (4.51)	0.070
***TXNIP* DNA methylation blood (N = 23/181)**	65.88 (5.67)	64.91 (7.32)	0.540
***TXNIP* gene expression SAT**^a^ **(N = 21/138)**	1.52 (0.78)	1.55 (0.91)	0.962
***TXNIP* gene expression skeletal muscle**^a^ **(N = 21/160)**	1.26 (0.46)	1.06 (0.47)	**0.038**

Data is mean (SD). Analysis of differences between groups was performed by independent samples t-test.

p-values < 0.05 are bold

N refers to number of subjects with abnormal OGTT vs. subjects with normal OGTT for each analysis

^a^ Data was log transformed prior to t-test.

Abbreviations: IFG, impaired fasting glucose; IGT, impaired glucose tolerance; T2DM, type 2 diabetes mellitus.

## Discussion

In this study, we were unable to confirm our a priori hypothesis of decreased *TXNIP* DNA methylation and increased gene expression in adult offspring of women with diabetes in pregnancy compared to controls. In contrast, we found an unexpected increased SAT *TXNIP* DNA methylation and a decreased *TXNIP* gene expression in the O-GDM subgroup. There were no significant differences in skeletal muscle or blood *TXNIP* DNA methylation or skeletal muscle gene expression between groups.

### *TXNIP* DNA methylation in blood

Although the differences were not statistically significant, the exposure groups in our cohort tended to have decreased blood *TXNIP* DNA methylation and blood *TXNIP* DNA methylation was significantly negatively associated with offspring fasting plasma glucose and insulin and HOMA-IR (Table 3). This is in line with previous findings of an inverse association between blood *TXNIP* DNA methylation levels and fasting plasma glucose and Hba1c levels, and between blood *TXNIP* DNA methylation and risk of T2DM [14–16, 18, 19], as well as decreased blood *TXNIP* DNA methylation in subjects with T2DM compared to controls [17]. While most of these studies included between 1,100 and 20,000 participants, of whom between 200 and 1,900 had T2DM, there were only a total of 23/206 (11%) subjects with abnormal glucose tolerance or T2DM in our study (O-GDM, n = 11; O-T1DM, n = 9; O-BP, n = 3), with the majority of participants having normal glucose tolerance. Thus, the lack of difference in *TXNIP* blood DNA methylation between exposure and control groups could be due a type 2 error, since we observed an expected trend of decreased methylation in blood in both exposure groups.

### *TXNIP* DNA methylation in tissues

To date, only one other study has examined *TXNIP* DNA methylation in tissues besides blood, finding decreased *TXNIP* DNA methylation in skeletal muscle and pancreatic islets, but no difference in adipose tissue *TXNIP* DNA methylation, in subjects with T2DM compared to controls [17]. By contrast, we found increased SAT *TXNIP* DNA methylation and no difference in skeletal muscle *TXNIP* DNA methylation in subjects predisposed to T2DM via fetal programming. These results, contrasting our a priori hypothesis, therefore do not suggest that altered *TXNIP* DNA methylation levels in SAT and skeletal muscle are responsible for the metabolic impairment observed in offspring exposed to maternal diabetes.

The increased SAT *TXNIP* DNA methylation in O-GDM in our cohort is furthermore in contrast to the findings of *TXNIP* blood and skeletal muscle hypomethylation with increasing Hba1c levels, increased risk of T2DM and overt T2DM [14–18], but the different tissues studied could explain the different results. Interestingly, in a post hoc subject stratification, we found decreased *TXNIP* SAT DNA methylation in subjects with abnormal glucose tolerance compared to those with normal glucose tolerance, similar to previous findings in blood and skeletal muscle. Although speculative due to the post hoc explorative subgroup analysis, this does provide some evidence that ambient plasma glucose levels, and not exposure to maternal diabetes, could be responsible for changes in *TXNIP* DNA methylation.

### *TXNIP* gene expression

The decreased SAT *TXNIP* expression in the exposure groups differs from a previous study which demonstrated increased *TXNIP* expression in cultured human adipocytes in response to glucose [22]. However, comparison between this study and our results is difficult because the in vitro effect of glucose on cells in culture cannot be directly extrapolated to gene expression levels in human biopsies. One study found that *TXNIP* knockout mice were protected against insulin resistance when challenged with a high-fat diet, remaining more insulin-sensitive than controls despite gaining more adipose tissue mass due to increased rates of insulin-stimulated glucose uptake in both skeletal muscle and adipose tissue [31]. Another study showed that *TXNIP* expression is inversely correlated with glucose-uptake in insulin-sensitive cells and tissues including adipocytes and skeletal muscle [22]. The lower SAT *TXNIP* gene expression levels could therefore be a compensatory mechanism that contributes toward slowing the natural disease process by increasing peripheral glucose uptake and maintaining insulin sensitivity in subjects at increased risk of future T2DM. This is supported by findings of no difference in adipose tissue *TXNIP* expression in subjects with T2DM compared to controls [17], indicating a lack of these compensatory mechanisms in subjects with overt disease. The similar findings of decreased *TXNIP* SAT expression in both O-GDM and O-T1DM (although only near-significant for O-T1DM) lend added credibility to our results, but studies of *TXNIP* expression in SAT are scarce, and we cannot exclude that our findings could be part of an as yet unknown pathogenic mechanism.

Studies have demonstrated decreased skeletal muscle and blood *TXNIP* DNA methylation and increased skeletal muscle *TXNIP* expression in subjects both at increased risk of as well as overt T2DM, and hyperglycemia increases *TXNIP* expression in various cells and tissues [17, 18, 22]. Therefore, we examined differences in *TXNIP* DNA methylation and expression in the 23 offspring with abnormal glucose tolerance (impaired fasting glucose IFG, impaired glucose tolerance IGT, or T2DM) compared to those with normal glucose tolerance. The increased skeletal muscle *TXNIP* gene expression (and borderline reduced *TXNIP* DNA methylation) in the subgroup of 23 offspring with abnormal glucose tolerance is in support of previous findings of increased skeletal muscle *TXNIP* expression in subjects with prediabetes and diabetes [22]. The lack of difference in skeletal muscle *TXNIP* expression in exposed offspring in our cohort could therefore be due to the fact that the majority of our subjects showed normal glucose tolerance and similar fasting blood glucose levels. These results thereby suggest that changes in SAT and skeletal muscle *TXNIP* DNA methylation and gene expression are more likely to be mediated by plasma glucose levels than to be a consequence of fetal programming.

### Tissue-specific differences in correlation between *TXNIP* gene expression and offspring parameters of glucose and insulin sensitivity

Interestingly, correlations between *TXNIP* expression and parameters of glucose and insulin sensitivity (fasting and 2-hour OGTT plasma glucose and insulin levels, HbA1c, HOMA-IR and total body fat percent) were tissue specific and opposite for SAT vs. skeletal muscle, with overall negative correlations in SAT but positive correlations in skeletal muscle. Thus, our results indicate tissue-specific differences in *TXNIP* expression that make it difficult to extrapolate information between different tissue types. These tissue-specific differences also provide a possible explanation for findings in other studies of decreased *TXNIP* DNA methylation and increased expression in skeletal muscle to be associated with T2DM, while we found increased SAT *TXNIP* DNA methylation and decreased gene expression in O-GDM in our cohort.

### Exposure to diabetes in pregnancy

O-T1DM are likely exposed to higher blood glucose levels in pregnancy, as evidenced by the fact that a larger proportion of O-T1DM were born large for gestational age compared to O-GDM [24], and the fact that GDM mothers in our cohort were diet-treated only. However, the greater difference in both methylation and gene expression in SAT for O-GDM suggest a role for factors besides maternal glucose levels alone playing a role on offspring metabolic phenotype. These factors include genes (O-GDM are expected to have greater genetic predisposition to T2DM), lifestyle, social and environmental factors, as well as potential intrauterine metabolites besides glucose (eg. amino acid or fatty acids).

The increased SAT *TXNIP* DNA methylation in O-GDM disappeared after adjustment for the offspring´s ambient insulin resistance (HOMA-IR), HbA1c levels and total body fat% (model 2, see Results section). However, SAT and muscle *TXNIP* functions are suspected to regulate tissue and whole body glucose uptake. Thus, cause-effect relationships between tissue *TXNIP* measurements on one side, and HbA1c, HOMA-IR and body fat % on the other, are unclear, why no clear conclusions can be drawn from changes in tissue *TXNIP* measurements after correction for the offspring´s ambient HbA1c, HOMA-IR and total body fat % levels.

### Strengths and limitations

The strength of our study lies in the availability of simultaneous information regarding methylation and gene expression in adipose tissue, skeletal muscle and blood from a relatively large, unique cohort of adult offspring exposed to maternal hyperglycemia. Especially in SAT and skeletal muscle, data regarding *TXNIP* DNA methylation is scarce and this study provides some of the first available knowledge about *TXNIP* DNA methylation levels in these tissues. Additionally, our sample size is relatively large, as many epigenetic studies investigating gene expression and DNA methylation in metabolically active tissues such as skeletal muscle and SAT include about 20 subjects in each group [32, 33].

Our study is performed on adult offspring, and the study design does not allow us to establish causality; Furthermore, we cannot say with certainty whether the absolute differences in average DNA methylation and gene expression observed between the exposure groups and the control group are a consequence of intrauterine exposure to maternal diabetes or a consequence of other factors pertaining to lifestyle and genetics that we cannot account for. However, the fact that the study includes adult offspring can also be viewed as a potential strength, as there are very few other studies examining epigenetic changes in adult offspring exposed to maternal diabetes, and the findings may indicate that epigenetic changes occurring as a result of detrimental fetal exposures have the potential to last a lifetime.

The participation rate from the first to second follow-up, between which there was approximately a 10-year gap, was 45% (206/456 = 45%), and 25% (206/812 = 25%) from the original cohort. Although a potential weakness, this is also a well-known limitation to studies where cohorts are followed over long periods of time. Moreover, there was a certain selection bias between the two rounds of follow-up as many subjects with impaired glucose metabolism in the first follow-up declined participation in the second follow-up, as described previously [24]. Our cohort is thus essentially composed of the healthiest subjects from the original cohort—however, this would tend to push towards an underestimation of the results. Finally, many comparisons have been performed without correction for multiple testing and with all significance levels provided as nominal significance, potentially increasing the risk of type 1 errors.

## Conclusion

In conclusion, our results suggest that decreased *TXNIP* DNA methylation and increased gene expression are unlikely to represent major pathogenic mechanisms in fetal programming of metabolic disease caused by exposure to maternal diabetes. Further studies are needed to confirm our unexpected findings of increased *TXNIP* DNA methylation as well as decreased *TXNIP* gene expression in the subgroup of O-GDM.

## Supporting information

S1 Table Correlations between *TXNIP* DNA methylation, expression, and clinical variables in offspring of women with gestational diabetes (O-GDM), offspring of women with type 1 diabetes (O-T1DM) and offspring of women from the background population (O-BP). Correlations are presented as Spearmans rank coefficient R (p-value) unless otherwise indicated. P-values <0.05 are bold. ^a^Pearsons rank coefficient. SAT: subcutaneous adipose tissue HOMA-IR: homeostatic model assessment insulin resistance.
(DOCX)
