# Supplementary material for: DNA methylation and gene expression of TXNIP in adult offspring of women with diabetes in pregnancy
Source: PLoS One. 2017 Oct 27;12(10):e0187038. doi: 10.1371/journal.pone.0187038 (PMC5659766; doi:10.1371/journal.pone.0187038)
Supplement: S1 Table — Correlations are presented as Spearmans rank coefficient R (p-value) unless otherwise indicated. P-values <0.05 are bold. aPearsons rank coefficient. SAT: subcutaneous adipose tissue HOMA-IR: homeostatic model assessment insulin resistance. (DOCX) [file pone.0187038.s001.docx]

**S1 Table: Correlations between *TXNIP* methylation, expression, and clinical variables in offspring of women with gestational diabetes (O-GDM), offspring of women with type 1 diabetes (O-T1DM) and offspring of women from the background population (O-BP).**

| **METHYLATION** | | | | | | | | | | **GENE EXPRESSION** | | | | | |
| --- | --- | --- | --- | --- | --- | --- | --- | --- | --- | --- | --- | --- | --- | --- | --- |
| ***Offspring data*** | ***TXNIP* DNA methylation SAT** | | | ***TXNIP* DNA methylation skeletal muscle** | | | ***TXNIP* DNA methylation blood** | | | ***TXNIP* gene expression SAT** | | | ***TXNIP* gene expression skeletal muscle** | | |
|  | **O-GDM**  **N=53** | **O-T1DM**  **N=49** | **O-BP**  **N=35** | **O-GDM**  **N=62** | **O-T1DM**  **N=62** | **O-BP**  **N=41** | **O-GDM**  **N=82** | **O-T1DM**  **N=65** | **O-BP**  **N=57** | **O-GDM**  **N=58** | **O-T1DM**  **N=59** | **O-BP**  **N=42** | **O-GDM**  **N=76** | **O-T1DM**  **N=63** | **O-BP**  **N=42** |
| **Methylation SAT** |  |  |  | **0.348 (0.024)^a^** | **0.299 (0.046)^a^** | 0.123 (0.510)^a^ | 0.189 (0.176)^a^ | -0.029 (0.846)^a^ | -0.088 (0.617)^a^ | 0.074 (0.683) | -0.014 (0.926) | 0.075 (0.698) |  |  |  |
| **Methylation skeletal muscle**  **muscle** | **0.348**  **(0.024)^a^** | **0.299 (0.046)^a^** | 0.123 (0.510)^a^ |  |  |  | **0.332 (0.008)^a^** | 0.025 (0.850)^a^ | **0.352 (0.024)^a^** |  |  |  | -0.229 (0.073) | -0.118 (0.362) | -0.080 (0.628) |
| **Methylation blood** | 0.189 (0.176) | -0.029 (0.846)^a^ | -0.088 (0.617)^a^ | **0.332 (0.008)^a^** | 0.025 (0.850)^a^ | **0.352 (0.024)^a^** |  |  |  |  |  |  |  |  |  |
| **Gene expression SAT** | 0.074 (0.683) | -0.014 (0.926) | 0.075 (0.698) |  |  |  |  |  |  |  |  |  | -0.063 (0.653) | -0.038 (0.777) | -0.202 (0.237) |
| **Gene expression skeletal muscle** |  |  |  | -0.229  (0.073) | -0.118 (0.362) | -0.080 (0.628) |  |  |  | -0.063 (0.653) | -0.038 (0.777) | -0.202 (0.237) |  |  |  |
| **Fasting plasma glucose** | -0.205 (0.141) | -0.116 (0.428) | 0.265 (0.124) | -0.180 (0.160) | -0.087 (0.503) | -0.132 (0.416) | -0.140 (0.211) | -0.138 (0.273) | -0.246 (0.068) | -0.200 (0.133) | -0.056 (0.672) | -0.260 (0.101) | 0.126 (0.279) | 0.063 (0.622) | -0.130 (0.418) |
| **2-hour plasma glucose** | 0.211 (0.146)^a^ | -0.177 (0.229)^a^ | **-0.378 (0.027)**^a^ | 0.245 (0.068)^a^ | 0.038 (0.769)^a^ | -0.217 (0.185)^a^ | 0.099 (0.395)^a^ | 0.109 (0.389)^a^ | -0.173 (0.205)^a^ | **-0.280 (0.044)** | 0.060 (0.654) | -0.257 (0.109) | **0.265 (0.027)** | 0.229 (0.073) | **0.363 (0.021)** |
| **HbA1C IFCC (mmol/mol)** | -0.050 (0.724)^a^ | -0.226 (0.118)^a^ | -0.022 (0.898)^a^ | -0.079 (0.541)^a^ | 0.007 (0.956)^a^ | 0.029 (0.860)^a^ | -0.016 (0.884)^a^ | -0.181 (0.150)^a^ | -0.122  (0.371)^a^ | **-0.282 (0.032)** | -0.020 (0.881) | -0.265 (0.095) | -0.129 (0.265) | **0.264 (0.036)** | 0.051 (0.750) |
| **HOMA-IR** | -0.234 (0.105) | 0.040 (0.797) | 0.141 (0.441) | -0.212 (0.123) | -0.005 (0.972) | -0.109 (0.528) | **-0.312 (0.007)** | 0.051 (0.700) | -0.208 (0.152) | **-0.425 (0.002)** | **-0.368 (0.006)** | **-0.508 (0.002)** | **0.251 (0.039)** | **0.275 (0.037)** | 0.293  (0.087) |
| **Fasting insulin (pmol/l)** | -0.231 (0.110) | 0.065 (0.674) | 0.048 (0.793) | -0.187 (0.176) | 0.004 (0.976) | -0.129 (0.447) | **-0.310 (0.007)** | 0.062 (0.637) | -0.157 (0.277) | **-0.401 (0.003)** | **-0.431 (0.001)** | **-0.445 (0.006)** | **0.252 (0.038)** | 0.254 (0.054) | 0.278 (0.101) |
| **Total body fat (%)** | **-0.300 (0.031)**^a^ | 0.008 (0.957)^a^ | -0.229 (0.186)^a^ | -0.169 (0.194)^a^ | 0.043 (0.741)^a^ | -0.054 (0.737)^a^ | -0.006 (0.956)^a^ | 0.146 (0.244)^a^ | -0.087 (0.521)^a^ | **-0.408 (0.002)** | -0.199 (0.131) | -0.244 (0.120) | **0.245 (0.034)** | **0.415 (0.001)** | **0.425 (0.005)** |

Correlations are presented as Spearmans rank coefficient R (p-value) unless otherwise indicated. P-values <0.05 are bold.

^a^Pearsons rank coefficient.

SAT: subcutaneous adipose tissue

HOMA-IR: homeostatic model assessment insulin resistance

SAT: subcutaneous adipose tissue
